# “Reprogram Enablement” as an Assay for Identifying Early Oncogenic Pathways by Their Ability to Allow Neoplastic Cells to Reacquire an Epiblast State

**DOI:** 10.1016/j.stemcr.2020.07.016

**Published:** 2020-08-13

**Authors:** Yanjun Kong, Ryan C. Gimple, Rachael N. McVicar, Andrew P. Hodges, Jun Yin, Yang Liu, Weiwei Zhan, Evan Y. Snyder

**Affiliations:** 1 Department of Imaging and Nuclear Medicine, Ruijin Hospital, School of Medicine, Shanghai Jiao Tong University, Shanghai, China; 2 Sanford Burnham Prebys Medical Discovery Institute, La Jolla, CA, USA; 3 Sanford Consortium for Regenerative Medicine, La Jolla, CA, USA; 4 Division of Regenerative Medicine, Department of Medicine, University of California, San Diego, CA, USA; 5 Department of Pediatrics, University of California, La Jolla, CA, USA

**Keywords:** tumor, cancer, human induced pluripotent stem cells, human embryonic stem cells, epiblast, reprogramming, RAS, oncogenesis, cancer stem cells, organoids, reprogram enablement

## Abstract

One approach to understanding how tissue-specific cancers emerge is to determine the requirements for “reprograming” such neoplastic cells back to their developmentally normal primordial pre-malignant epiblast-like pluripotent state and then scrutinizing their spontaneous reconversion to a neoplasm, perhaps rendering salient the earliest pivotal oncogenic pathway(s) (before other aberrations accumulate in the adult tumor). For the prototypical malignancy anaplastic thyroid carcinoma (ATC), we found that tonic RAS reduction was obligatory for reprogramming cancer cells to a normal epiblast-emulating cells, confirmed by changes in their transcriptomic and epigenetic profiles, loss of neoplastic behavior, and ability to derive normal somatic cells from their “epiblast organoids.” Without such suppression, ATCs re-emerged from the clones. Hence, for ATC, RAS inhibition was its “reprogram enablement” (RE) factor. Each cancer likely has its own RE factor; identifying it may illuminate pre-malignant risk markers, better classifications, therapeutic targets, and tissue-specification of a previously pluripotent, now neoplastic, cell.

## Highlights

• The factors for reprogramming a cancer cell to an epiblast-like cell can be assayed
• “Reprogram enablement” can yield insights into the earliest pivotal oncogenic steps
• For anaplastic thyroid carcinoma, RAS inhibition was obligatory for reprograming
• Each tissue-specific cancer will have its own reprogramming enablement requirement

## Teaser

In this article, Snyder and colleagues show that the minimal essential factor(s) needed to convert a cancer cell back to its normal, pre-malignant, epiblast-like pluripotent state and then back again into a neoplastic cell—its “reprogram enablement factor”—may provide insights into the earliest pivotal steps in that cancer's tissue-specific oncogenesis, as well as its early diagnosis and treatment.

## Introduction

The human embryonic stem cell (ESC), obtained by culturing the inner cell mass (ICM) of a blastocyst, is regarded as our best *in vitro* model of the human epiblast. There are strategies for emulating an ESC by dedifferentiating—or “reprogramming”—an ostensibly end-differentiated somatic cell. The first example of reprogramming entailed placing the nucleus of a somatic cell (e.g., a dermal fibroblast) into the cytoplasm of an enucleated fertilized oocyte—a process called somatic cell nuclear transfer (Gurdon, 1962). Four decades later, it was demonstrated that fibroblasts could be reprogrammed into ESC “mimics”—called induced pluripotent stem cells (iPSCs)—by introducing into them the minimal essential transcription factors associated with the ESC-like state—*Oct4*, *Sox2*, *Klf4*, and *Myc* (OSKM) (Yamanaka, 2012). Human iPSCs (hiPSCs) are derived from somatic cells that have “lost” their cell-type identity and come to resemble, molecularly and functionally, the pluripotent human ESC (hESC). Importantly, the hiPSCs retain some of the genetic fingerprint—for example, mutations—of the starting somatic cell. Hence, if the starting cell is obtained from a patient with a given disease (particularly one that is genetically based), the hiPSC derived from that somatic cell will also still “have” that disease, but in a setting amenable to scrutiny and experimental manipulation. Studying such hiPSCs can provide insights into cell-type specification and plasticity, disease pathogenesis, as well as offer tools for drug discovery. We hypothesized that, hiPSCs derived from a cancer cell would similarly maintain the propensities accumulated in that neoplastic cell's genome (including mutations) and enable us to ask a number of intriguing questions: (1) What are the progressive steps and requirements for transiting from a neoplastic cell to a developmentally “normal” cell and back again to a neoplastic one, starting at the primordial pre-malignant stages of embryogenesis, perhaps throwing into relief a pivotal early genetic pathway gone awry (before the many that may ultimately be present in the fully formed adult tumor)? (2) Why and how does a given neoplastic cell acquire a particular tissue identity as it transits from a “pluripotent” state (where an “oncogene” could influence any cell or organ) to “lineage commitment” (i.e., producing a cancer of a particular cell type in a particular organ—for example, why cancer of the thyroid and not of the brain)? Do mutations in the hiPSCs “inherited” from the original cancer cells still function in a tissue-specific manner and how? (3) Do any therapeutic targets exist that might be attacked at early pre-malignant stages and/or are there biomarkers that might be used for early pre-morbid cancer risk assessment or diagnosis? In other words, the ability to observe the transition from a neoplastic cell back into a normal cell in the “epiblast” and then back again into a neoplastic cell may offer insights into novel anti-cancer therapies (Kim and Zaret, 2015; Lang et al., 2013; Lee et al., 2015; Stricker and Pollard, 2014; Iskender et al., 2016; Herreros-Villanueva et al., 2013). Established cancer cell lines for such studies have been useful in the past but, unfortunately, are susceptible to additional genetic and epigenetic changes during prolonged culturing that might not be directly related to oncogenesis and hence provide confounding data. In “starting from scratch,” tumor-derived hiPSCs may circumvent this complication.

To serve as a prototypical extremely malignant neoplasm, we chose anaplastic thyroid carcinoma (ATC). No treatment significantly improves prognosis (median survival <6 months) because of its refractoriness to chemotherapy and radiotherapy (Lee et al., 2016). Accumulation of genetic and epigenetic alterations and their consequent derangements in downstream signaling appear to be key to ATC's pathogenesis (Xing, 2013). In particular, the T1799A transverse point mutation in the *BRAF* gene results in a mutant BRAF-V600E protein with a constitutively active serine/threonine kinase, which, in turn, promotes tumorigenesis, invasion, metastasis, recurrence, and mortality.

Generating hiPSCs from normal somatic cells has become fairly routine. It would seem to be equally uncomplicated to use the same successful protocols to reprogram a neoplastic cell into an hiPSC. However, making tumor-derived hiPSCs is not as straightforward as might be assumed. Others have also encountered challenges in reprogramming cancer cells and agree that it is the most difficult reprogramming cases that will prove the most instructive (Zhao et al., 2015; Camara et al., 2016). Hence, here we share both our failure and then the requirements for successful creation of hiPSCs from this virulent malignancy, highlighting how such findings may provide insights into the earliest oncogenic process as well as its therapy. In this proof-of-concept instance, we found that successful reprograming of human ATC back into normal human epiblast-emulating cells (at the transcriptional, epigenetic, protein, and functional levels) was dependent on suppressing RAS signaling. Without such inhibition, thyroid cancer (although not another RAS-related tumor, such as melanoma) re-emerged spontaneously without priming from this “pre-malignant” normal epiblast-like developmental state (a process which itself can be scrutinized in an unbiased “naturalistic” manner).

The National Cancer Institute has made the identification of next-generation cancer models (NGCMs) a priority. We suggest here that “developmental regression” to a normal pre-malignant epiblast-like state—an assay that might be termed reprogram enablement—may help provide one of those models.

## Results

### ATC Cells Did Not Express Pluripotency Genes

For reprogramming, we chose four representative histopathologically categorized ATC cell lines originally obtained from adult patients: C643, Hth74, 8505C, and SW1736 (see Supplemental Information). Notably, the latter two have a mutation in the Ras pathway—i.e., the V600E locus in the *BRAF* gene—whereas the former two are normal (wild type) in that locus (a point to which we will return in the Discussion).

**Figure 1:**
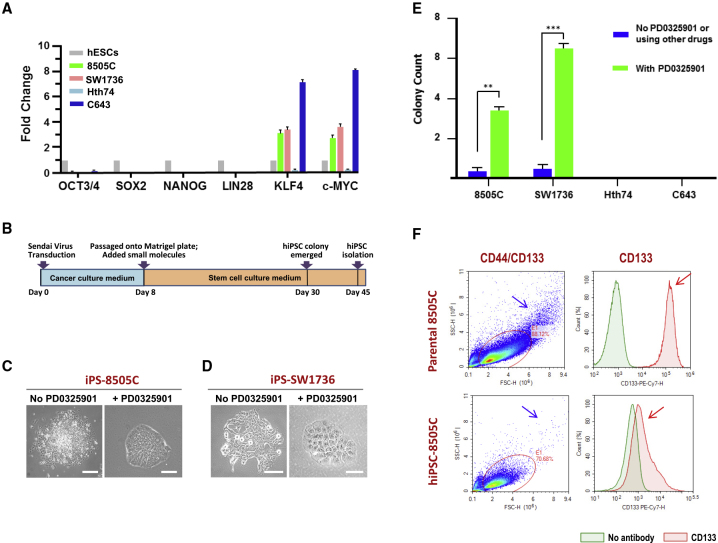
Reprogramming Virulent ATC Cells into hiPSCs (A) Stemness-related gene expression of cancer cells compared with hESCs, the gold standard of epiblast-modeling cells. Gene expression levels in the cancer cells were normalized to that in the hESCs. The expression levels of pluripotency markers *OCT-3/4*, *SOX2*, *NANOG*, and *LIN28* were virtually undetectable in all four parental cancer lines compared with their levels in hESCs; the findings were the same for the other standard pluripotency markers SSEA-4, TRA-1-60, and TRA-1-81, shown by immunocytochemistry in Figure S3. By contrast, expression of the oncogenes *KLF4* and *cMYC* was ∼2–3 times higher in both the 8505C and SW1736 ATC lines and 7–8 times higher in C643 compared with their levels in hESCs; only Hth74 had *KLF4* and *cMYC* expression levels lower than in hESCs. Three independent experiments were performed. (B) Schematic of the Sendai viral reprogramming procedure. See Figure S1 for efficiency of episomal vector transfection. (C and D) Colony morphology of a cancer-derived hiPSC is more like that of an hESC with tonic RAS suppression. Scale bars, 100 μm. (E) Comparison of the number of hiPSC colonies formed for each cancer-derived hiPSC without and with Ras suppression. RAS pathway inhibition not only improves colony morphology but also increases the number of hESC-like colonies. The result was reproducible in three independent experiments. (F) Cancer stem cells (CSCs) present in the parental ATC lines are not simply selected and expanded by the reprogramming process. Representative flow cytometry data are shown in two different formats for the well-accepted CSC markers CD133 and CD44 for the representative ATC line 8505c. The upper panels show CSC marker expression by the parental cancer population. The lower panels show expression by the ATC-derived hiPSC population. The right panels, assay for expression of CD133. Note that a red CD133 peak is present in the cancer population (red arrow) but is indistinguishable from the green negative control peak in the hiPSC population. The left panels, assay for cells with dual expression of CD133 and CD133. The cancer cell population has a prominent dual-positive bin (blue arrow) which largely disappears in the cancer-derived hiPSC population (blue arrow). All data were collected from three independent experiments. Error bars indicate the standard error of the mean. Related to Figures S1–S3

We first determined the expression of known oncogenes and pluripotency genes in the ATCs before reprogramming. The baseline expression levels of these pluripotency markers were essentially absent in all four cancer cell lines when compared with their levels in hESCs (Figure 1A). We then checked the expression levels of *KLF4* and *cMYC*, both of which are important for the induction of pluripotency but are also very prominent oncogenes. The expression of *KLF4* and *cMYC* was approximately three to four times higher in ATC lines 8505C and SW1736 and seven to nine times higher in C643 compared with their levels in hESCs; only Hth74 had expression levels of *KLF4* and *cMYC* lower than in hESCs (Figure 1A). These findings are consistent with the view that pluripotency and neoplasia share many molecular characteristics—in some cases, distinguished by degree not by absolute presence or absence.

### Sendai Virus-Mediated Transduction of Reprogramming Factors Enabled the Generation of Transient hiPSCs from ATC Cells Bearing a Ras Mutation, but Not from Those without

The success and efficiency of hiPSC generation depend on a number of features of the starting cell, not all of which are yet known by the field. Those factors that are known include proliferative capacity and genetic background of the starting cell, and method of delivery of reprogramming factors. In this study, two non-integrating gene transfer methods were tested on all four ATC lines: episomal and Sendai virus (SeV) vectors.

Episomal vectors carrying the OSKM reprogramming factors were electroporated into the ATC cells (Tobe et al., 2017). Transfection efficiency was 30%, as determined by co-transfecting a vector expressing green fluorescence protein (Figure S1). Despite successful transfection, no hiPSC colonies were observed in any of the ATC lines after four independent trials.

SeV-mediated reprogramming was then applied (Figure 1B). hiPSC colonies emerged after 30 days from Ras-mutated ATC lines 8505C (called iPS-8505C) (Figures 1C and S2) and SW1736 (called iPS-SW1736) (Figures 1D and S2), but not from the two non-Ras-mutated ATC lines, C643 and Hth74 (Figure S3). SeV-related proteins were undetectable in post-reprogrammed hiPSCs by passage 10, as confirmed by immunocytochemistry (data not shown). Reprogramming efficiency was ∼0.2%, considered acceptable given that it falls within the 1%–0.01% range typically reported for non-neoplastic normal somatic cells. However, these colonies could not be sustained.

### Tonic RAS Pathway Inhibition Was Required for Generating and Maintaining hiPSCs Derived from ATCs

To enhance reprogramming efficiency and generate stable hiPSC clones, we explored multiple additional methods, including inhibiting (1) Rho/ROCK signaling (with Y-27632, a Rho-associated, coiled-coil containing protein kinase [ROCK] inhibitor); (2) transforming growth factor β (TGF-β) signaling (with SB431542, a TGF-β-R1 [ALK5] inhibitor); (3) Wnt signaling (with CHIR99021, a glycogen synthase kinase-3β inhibitor); and (4) RAS/MAPK signaling (with PD0325901, a mitogen-activated protein kinase [MEK] inhibitor) (see Supplemental Information). These small molecules were tested at different concentrations and in different combinations as detailed in Supplemental Information on all four ATC cell lines. The colonies that emerged following RAS inhibition (using PD0325901 for 7 days post-SeV-mediated transduction) most emulated those of hESCs (the “gold standard”) (Figures 1C and 1D). Inhibiting RAS also yielded significantly more such colonies (Figure 1E). Altering the other pathways with the above-mentioned small molecules had no effect on reprogramming. The reprogramming efficiency of ATC cell lines 8505C and SW1736 to hiPSCs—under tonic RAS inhibition—increased to 6% and 3%, respectively (at 25 days post-viral transduction). Compared with the above-stated 0.2% efficiency without PD0325901, these reprogramming efficiencies with PD0325901 represented a 30-fold increase for 8505C and a 15-fold increase for SW1736. This assessment was based on immunostaining for the pluripotency markers alkaline phosphatase, TRA-1-81, TRA-1-60, SSEA-4, OCT-4, SOX2, and NANOG (Figure S3). PD0325901 alone was not sufficient to enable reprogramming. Furthermore, continued tonic application of PD0325901 was required to sustain the colonies once generated and to allow them to re-form following passaging. These hiPSCs could be passaged repeatedly, certainly beyond the 15-passage threshold used by the field to deem an hiPSC line as stable. No colonies emerged from C643 and Hth74 (those without mutated BRAF) despite multiple attempts of using PD0325901 alone or SeV-mediated reprogramming combined with PD0325901 (Figure S2).

### Ruling out the Possibility that the hiPSCs Were Actually Enriched and Expanded Cancer Stem Cells

To exclude the possibility that what we were calling “cancer-derived hiPSCs” might actually represent simply an enrichment and expansion of the small population of highly tumorigenic cells known as cancer stem cells (CSCs) that populate many solid tumors (Ma et al., 2014; Nagayama et al., 2016; Todaro et al., 2010), we analyzed the parental cancer population and their respective reprogrammed RAS-inhibited hiPSCs for the most commonly accepted CSC markers, CD44 and CD133, using flow cytometry. The percentages of CD44^+^ and/or CD133^+^ cells in the hiPSC population were virtually non-existent compared with their respective parental cancer cell populations (Figure 1F). The absolute numbers and percentages of the sub-population of CD44^+^ and/or CD133^+^ cells (including dual-positive cells) were orders-of-magnitude less than in the ATC cell population. Furthermore, as noted below (e.g., Figures 3B and 3C), the gene expression profile, methylation landscapes, and principal component analysis of our thyroid cancer-derived hiPSCs cluster with hESCs, a profile very distinct from that of CSCs. Therefore, it was reasonable to conclude that the colonies designated hiPSCs were, indeed, pluripotent stem cells, distinct from CSCs and not merely a selection, expansion, and enrichment of the CSCs.

### Differentiation Potential of the Cancer Cell-Derived hiPSCs

**Figure 2:**
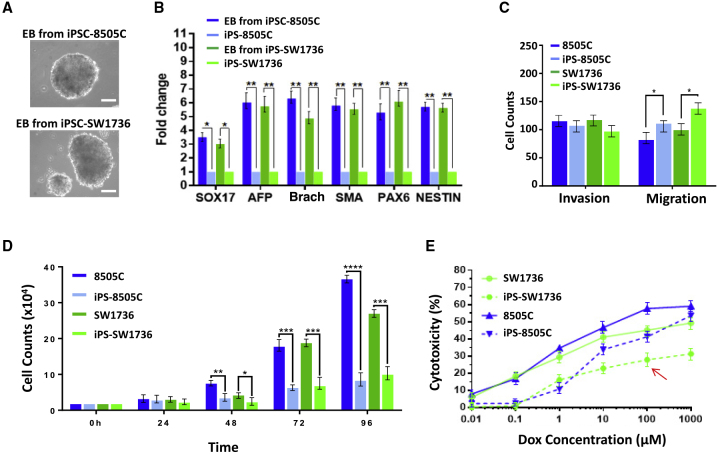
Properties and Differentiation Potential of the Cancer-Derived hiPSCs (A and B) Differentiation of hiPSCs into normal derivatives of all three germ layers within an epiblast “organoid” (also termed an embryoid body [EB]). (A) Phase photomicrograph of the EBs derived from iPS-8505C (upper panel) and from iPS-SW1736 (lower panel). Scale bars, 100 μm. (B) Gene expression levels of derivatives from the three fundamental germ layers which differentiated within EBs generated from the cancer-derived hiPSCs (iPS-8505C and iPS-SW1736) compared with the respective hiPSC in monolayer before EB formation (as measured by RT-PCR): endoderm markers *SOX17* and AFP (alpha-fetoprotein); mesoderm markers BRACHYURY (BRACH) and SMA (α-smooth muscle actin); ectoderm markers PAX6 and NESTIN. See Figure S4 for immunostaining for these markers. The result was reproducible in three independent experiments. (C–E) Some characteristics of malignancy in iPS-8505C and iPS-SW1736 compared with their respective starting parental cancer cells (8505C and SW1736). (C) Cell migration and invasion assays showed that the migratory ability of the hiPSCs increased (typical for a stem cell) compared with their respective parental cancer cells but without an increase in their invasiveness. These experiments were repeated at least three times. (D) Assessing proliferation based on cell counts over time showed that proliferation of the hiPSCs was significantly decreased compared with that of their parental cancer cells. The experiment was repeated at least three times. (E) Sensitivity to classic chemotherapeutic drugs of the parental cancer cells compared with their respective reprogramed hiPSCs, using an MTT-based assay to distinguish between cytostatic and cytotoxic responses. The slowly proliferative cancer cell-derived hiPSCs (especially iPS-SW1736 [red arrow]) lost much of their sensitivity to doxorubicin (doses between 0.01 and 1,000 μM), another sign of loss of a malignant neoplastic phenotype. The data were collected from three independent experiments. Error bars indicate the standard error of the mean. Related to Figure S4

Both iPS-8505C and iPS-SW1736 were able to form epiblast-modeling “organoids,” also called embryoid bodies (EBs), after 10 days of maintenance in EB formation medium (Figure 2A). There was no significant difference between the number of EBs formed by iPS-8505C versus iPS-SW1736. Differentiation into somatic cells representing all three fundamental germ layers (endoderm, mesoderm, and ectoderm) within the EBs was verified by immunocytochemistry (Figure S4) and by qRT-PCR (Figure 2B; see Supplemental Information). The field's routinely accepted lineage markers—alpha-fetoprotein (AFP) and SOX17 for endoderm; brachyury and α-smooth muscle actin for mesoderm; and PAX6 and Nestin for ectoderm (Pekkanen-Mattila et al., 2010; Lee et al., 2007)—were highly expressed in the hiPSC-derived EBs, confirming pluripotency by demonstrating the full differentiation potential of iPS-8505C and iPS-SW1736 in a manner similar to normal somatic cell-derived hiPSCs and hESCs.

Since it has been speculated that hiPSCs may be predisposed to differentiate back to the same tissue cell type from which they were derived (Kim et al., 2010), we determined whether iPS-8505C and iPS-SW1736 had a predilection for differentiating toward endoderm, the germ-layer-of-origin of thyroid. As seen in Figures 2B and S4, expression of the endoderm markers SOX17 and AFP within their EBs was no more prominent than that of the other germ layers.

**Figure 3:**
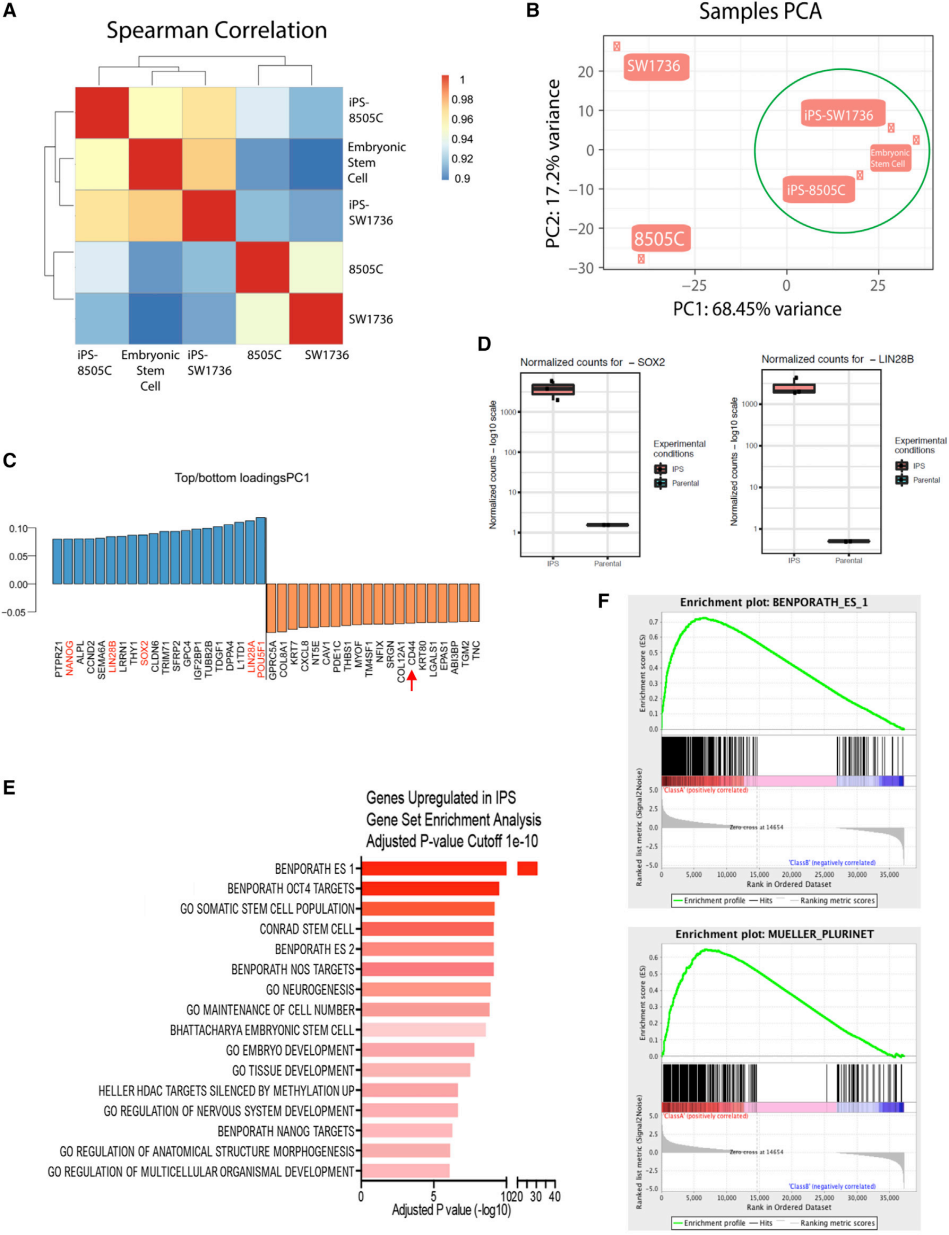
Transcriptional Programs Distinguish the Cancer-Derived hiPSCs from Their Respective Starting Cancer Cells; They Hew Closer to Gold Standard hESCs than to Their Neoplastic Parental Cells (A) Spearman correlation heatmap showing correlation between samples based on global transcriptional profiling by RNA-seq. hiPSCs (iPS-8505C and iPS-SW1736) derived from each cancer cell line (8505C and SW1736, respectively) were both better correlated with hESCs than with their starting cancer cells. (B) Principal component analysis (PCA) of global transcriptional profiling by RNA-seq. hiPSCs derived from each cancer cell line were in the same group with hESCs (green circle) (principal component 1 [PC1]); the two cancer cell lines segregated to a different group. (C) Top genes for PC1 shown in (B). Genes shown in blue are positively correlated with PC1, and contain many pluripotency genes (in red). Genes indicated in orange are negatively correlated with PC1 and, for example, contain a marker for cancer stem cells (CD44, red arrow). (D) Normalized transcript counts for the stemness genes *SOX2* and *LIN2*8B in the hiPSCs versus the parental cancer populations. Transcripts per million values from the RNA-seq data were compared with the mRNA expression of cancer cells before (parental) and after (hiPSC) induction of pluripotency. Sequencing reads were mapped to the genome using Salmon (Patro et al., 2017) and further processed using TXimport (Soneson et al., 2015) and DESEQ2 (Love et al., 2014) to generate normalized counts based on sequencing depth and gene length. As shown in this panel, *SOX2* and *LIN2*8B have elevated mRNA expression levels in the hiPSC group relative to their parental cancer group. (E) Gene set enrichment analysis for the top 100 genes upregulated in hiPSCs compared with parental populations at the mRNA level. The most upregulated pathways in the hiPSCs compared with the cancer parental cells were pathways associated with hESC pluripotency, stem cell maintenance, neurogenesis, embryo development, and tissue development. (F) Gene set enrichment analysis for the “BENPORATH ES 1” dataset (which includes genes overexpressed in hESCs [according to 5 or more out of 20 profiling studies]) (false discovery rate [FDR]-corrected p value <0.0001) and the “MUELLER PLURINET” dataset (which includes genes constituting the PluriNet protein-protein network shared by human pluripotent stem cells—hESCs, embryonical carcinomas, and hiPSCs) (FDR gene signatures). This analysis showed that the hiPSCs (group on the left side of the graph) were enriched in the two datasets while cancer cells (group on the right side of the graph) only expressed a few of the genes in the two datasets.

Classically, although not as common in today's literature, teratoma formation was regarded as the final test of pluripotency. We recognized, however, that such data would be difficult to interpret in this study because of the strong tendency of these ATC-derived hiPSCs to revert to their parental neoplastic state in the absence of tonic RAS suppression—an inhibition that cannot be locally maintained after their implantation *in vivo* subcutaneously or under the kidney capsule: any mass that formed would likely be dominated by thyroid cancer and obscure or even inhibit a teratoma. In other words, the reverted rapidly dividing ATC cells would overwhelm the slower-growing teratomas formed by the hiPSCs. Therefore, we used what has come to be accepted in the literature: noting spontaneous differentiation into, and gene expression indicative of, representative cell types comprising the three fundamental germ layers during EB formation (Figures 2B and S4). In addition, as detailed below, RNA sequencing (RNA-seq) (Figure 3) and methylation data (Figure 5) further attested to the similarity of the cancer-derived hiPSCs to gold standard pluripotent hESCs.

### hiPSC Reprogramming Altered the Cancer Phenotype

We next assessed the effects of reprogramming the ATCs to hiPSCs on their previous “cancer properties,” including proliferation, invasion, and sensitivity to the commonly used thyroid cancer chemotherapeutic drug doxorubicin.

The proliferation rate of the hiPSCs was much slower than that of the respective parental cancer cells (Figure 2D). Migration and invasiveness are often studied as surrogates for tumor aggressiveness and metastasis. Migration is also central to other cellular functions, including embryonic morphogenesis. Migration and invasion both entail cell movement, but the latter also requires the cells to penetrate a barrier-like extracellular matrix or basement membrane extract by first enzymatically degrading the barrier and then translocating to another spot. Our analyses of hiPSC lines iPS-8505C and iPS-SW1736 showed that their migratory ability increased compared with their respective parental cancer cells (consistent with being an “organogenic” stem cell) but without an increase in their invasiveness (a sign of malignancy and virulence) (Figure 2C).

Next, we compared the relative sensitivity of the parental cancer cells versus their respective reprogramed hiPSCs to a classic anti-thyroid cancer chemotherapeutic drug (doxorubicin) using a methyl thiazolyl tetrazolium (MTT)-based method to distinguish between cytostatic and cytotoxic responses. The slowly proliferative cancer-derived hiPSCs lost much of their sensitivity to doxorubicin (Figure 2E), another sign of losing a neoplastic phenotype (Kumano et al., 2012; Nagai et al., 2010).

### At the Transcriptional and Epigenetic Levels, the hiPSCs Were Closer to hESCs Than to Their Parental Cancers

**Figure 4:**
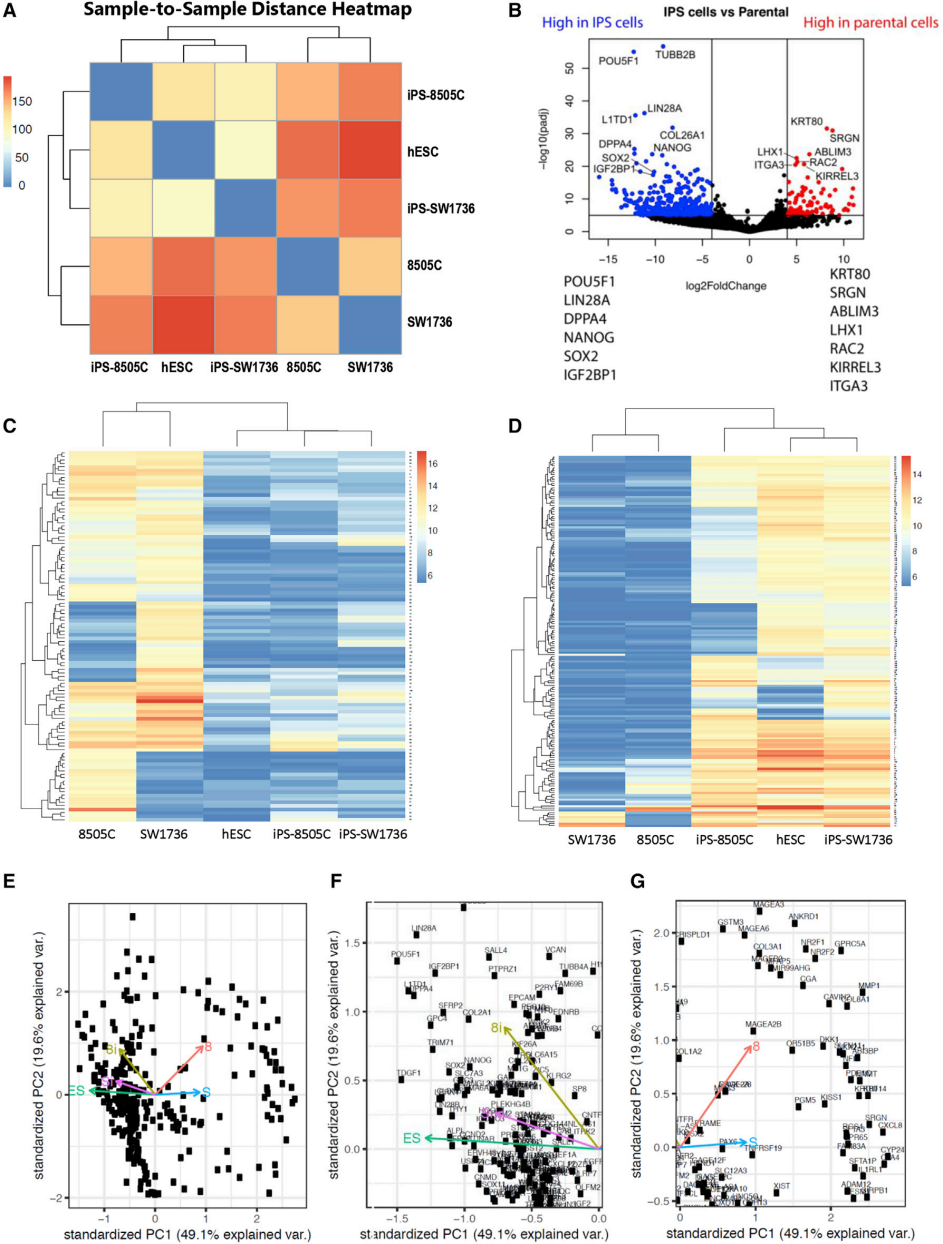
Cancer Cell-Derived hiPSCs and hESCs Have Similar Gene Expression Patterns (A) Sample-to-sample distance heatmap between samples based on global transcriptional profiling by RNA-seq showed that the gene expression of the hiPSCs derived from each cancer cell line (iPS-8505C and iPS-SW1736) was very similar to that of hESCs, but very different from that of the cancer starting cells (8505C and SW1736, respectively). (B) Volcano plot showing differentially expressed genes between hiPSCs and their respective parental cancer cells. Blue dots indicate genes upregulated in hiPSCs, while red dots indicate genes upregulated in parental cancer cells by RNA-seq analysis. The dots for some representative key genes, for example, those listed at bottom left and bottom right, are labeled. (C and D) Heatmaps showing genes downregulated (C) and upregulated (D) after hiPSC induction as measured by RNA-seq analysis showing that the hiPSC groups (iPS-8505C and iPS-SW1736) together with hESCs in both categories, and not with the parental cancer lines (8505C and SW1736, respectively). (Biocarta analysis of the RAS pathway alone in hESCs is provided in Figure 6A.) (E–G) Principal component analysis of the five cell lines from this study (parent ATC line 8595C and its reprogrammed hiPSC line iPS-8505C, ATC line SW1736 and its reprogrammed hiPSC line iPS-SW1736, and hESCs) from RNA-seq data overlaid upon each other (E). In (F) and (G), the information is segregated to allow better visualization. hiPSCs showed a similar gene expression pattern to hESCs (F) while the parental cancer cells showed similar gene expression patterns to each other but distinct from the hiPSCs and hESCs (G).

Having observed phenotypic changes in the reprogramming of cancer cells to hiPSCs in which the latter behaved more like normal human epiblast-modeling hESCs than ATCs, we next determined whether there were concomitant changes in the transcriptional and epigenetic profiles of the cancer cells after reprogramming. Therefore, we isolated RNA from 8505C and SW1736 cancer cells as well as from their reprogrammed hiPSC counterparts for whole-transcriptome profiling. hESCs were again used as the gold standard for pluripotency. Whole-transcriptome analysis by RNA-seq showed that the hiPSCs clustered together, close to hESCs, and distant from their parental counterparts (Figures 3A, 3B, and 4A). The genes that most clearly distinguished the hiPSCs from the parental cancers included well-known pluripotency markers, such as *POU5F1*, *LIN2*8A, *LIN2*8B, *SOX2*, and *NANOG* (Figures 3C and 4B), which were highly expressed in hiPSCs and hESCs, but not in the parental cancer cells (Figures 3D and 4B–4G); conversely, cancer-related genes, for example, those for CSCs (e.g., CD44), were downregulated in the hiPSCs (Figures 3C and 4C). Through unbiased analysis of the differentially expressed genes (Patro et al., 2017; Subramanian et al., 2005; Wang et al., 2017b) (Figures 3C and 3D), we found that the genes upregulated after reprogramming comprised pathways involved in hESC pluripotency, stem cell maintenance, neurogenesis, embryo development, and tissue development at a significance level of 1e-10 (Figures 3E and 3F). Taken together, these data suggest that the induction of pluripotency led to a reorganization of the global transcriptome to promote stem-like properties in the parental cancer cells.

**Figure 5:**
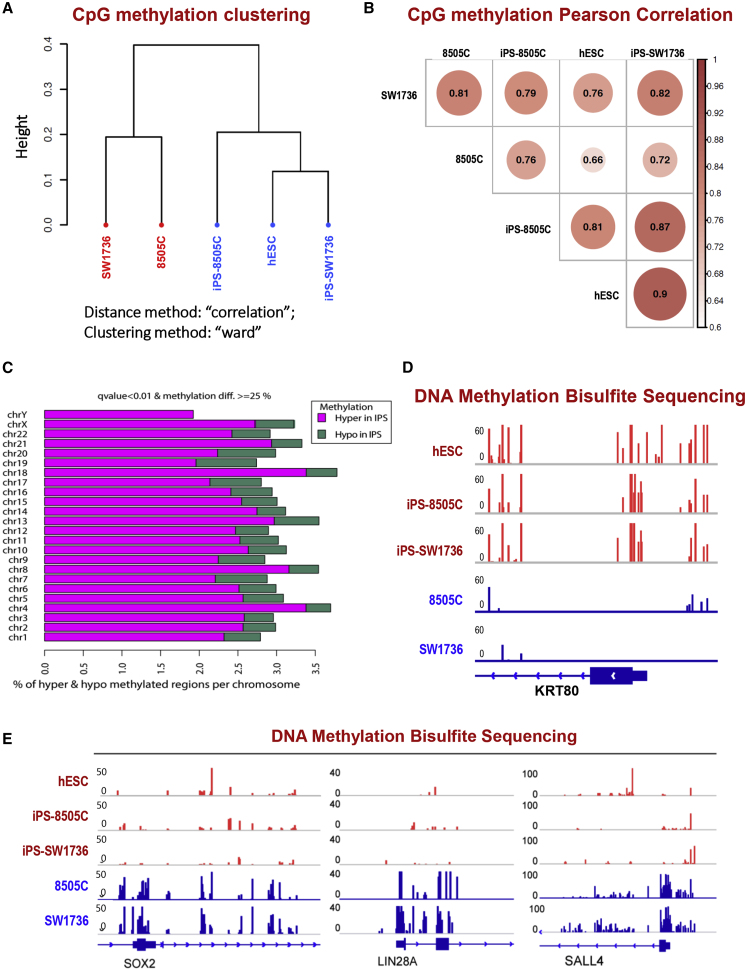
DNA Methylation Profiles Distinguish hiPSCs, which Emulate hESCs, from Their Starting Parental Cancer Cells (A) Sample clustering based on DNA methylation profiling by capture bisulfite sequencing. The Ward clustering method was used. hiPSCs (iPS-8505C and iPS-SW1736) and hESCs were in the same group and the two starting cancer cell lines (8505C and SW1736, respectively) were in another group. (B) Correlogram depicting sample similarity based on DNA methylation profiling. Pearson correlation was used to compare samples. hiPSCs and hESCs showed good correlation. (C) Plot showing differentially methylated regions by chromosome. To identify consistently significantly altered DNA methylation sites genome-wide, independent of clone or passage and to enhance signal-to-noise salience, the combined global methylation profiles from both iPS-8505C and iPS-SW1736 were compared with the combined parental cancer lines, 8505C and SW1736. More sites were hyper-methylated in the hiPSC group than in the parental cancer group. Pink bars indicate regions hyper-methylated in hiPSCs. Green bars indicate regions hypo-methylated in hiPSCs. q value <0.01 with a methylation difference of >25% were used for cutoffs. (D and E) DNA methylation genome track displaying bisulfite sequencing results surrounding (D) the *KRT80* locus and (E) the *SOX2*, *LIN2*8A, and *SALL4* loci (see Table S1). Related to Table S1.

Given that establishing pluripotency is known to require widespread epigenetic reconfiguration (Mahalingam et al., 2012; Stricker et al., 2013), we also analyzed the DNA methylation landscape of the cancer-derived hiPSCs. Pearson correlation statistics were used to demonstrate the unsupervised genome-wide concordance of methylation profiles across the genome (Figures 5A and 5B). In this study, we used multiple tests with a corrected false discovery rate (q value) instead of a p value (given that the latest thinking and standard practice in the field is that the q value is more appropriate for multiple test corrections when operating on genomic intervals). An example is shown in Table S1, which lists the differentially methylated regions along with the corresponding p value, q value, and methylation differences for *SOX2* and *SALL4*, genes associated with pluripotence and stem cell behavior. To identify consistently significantly altered DNA methylation sites genome-wide—independent of clone or passage—and to enhance signal-to-noise salience, the combined global methylation profiles from both iPS-8505C and iPS-SW1736 were compared with the combined parental cancer cells, 8505C and SW1736. In Figure 5C, we interrogated hyper- and hypo-methylated genomic regions and displayed those sites that had a methylation difference of >25% at a significance value (q value) of 0.01. As with the transcriptional analysis, the hiPSCs clustered together with the hESCs and away from their parental cancers (Figures 5A and 5B). These data suggested that treating the ATCs with reprogramming factors led to a reorganization of the epigenome toward a more normal stem-like state, emulating that of hESCs. Interestingly, induction of pluripotency led to hyper-methylation across the genome (Figure 5C). We interrogated the methylation profile of a few selected genes, predicting that it would be the cancer genes that are hyper-methylated and, hence, downregulated, while the pluripotency genes would be hypo-methylated and upregulated. That prediction appeared to be supported. For example, *KRT80* showed a consistent increase in DNA methylation surrounding the promoter (Figure 5D). Reciprocally, the genes upregulated in pluripotent cells (*SOX2*, *LIN2*8A, and *SALL4*) showed a decrease in DNA methylation surrounding their promoters (Figure 5E). The RNA-seq profile showed good correlation with the methyl sequencing profile in the arrow plots (Figure 4F) and projection plot (Figure 4G). Collectively, these findings suggested that the DNA methylation landscape of the cancer-derived hiPSCs was dramatically reorganized to sustain long term the epigenomic states that maintain pluripotency and suppress neoplasia.

### The Dependence on RAS Suppression for ATC's Developmental “Rewind” to a Normal Epiblast-like State Helped Pinpoint that Pathway as Pivotal in Early Thyroid Oncogenesis

**Figure 6:**
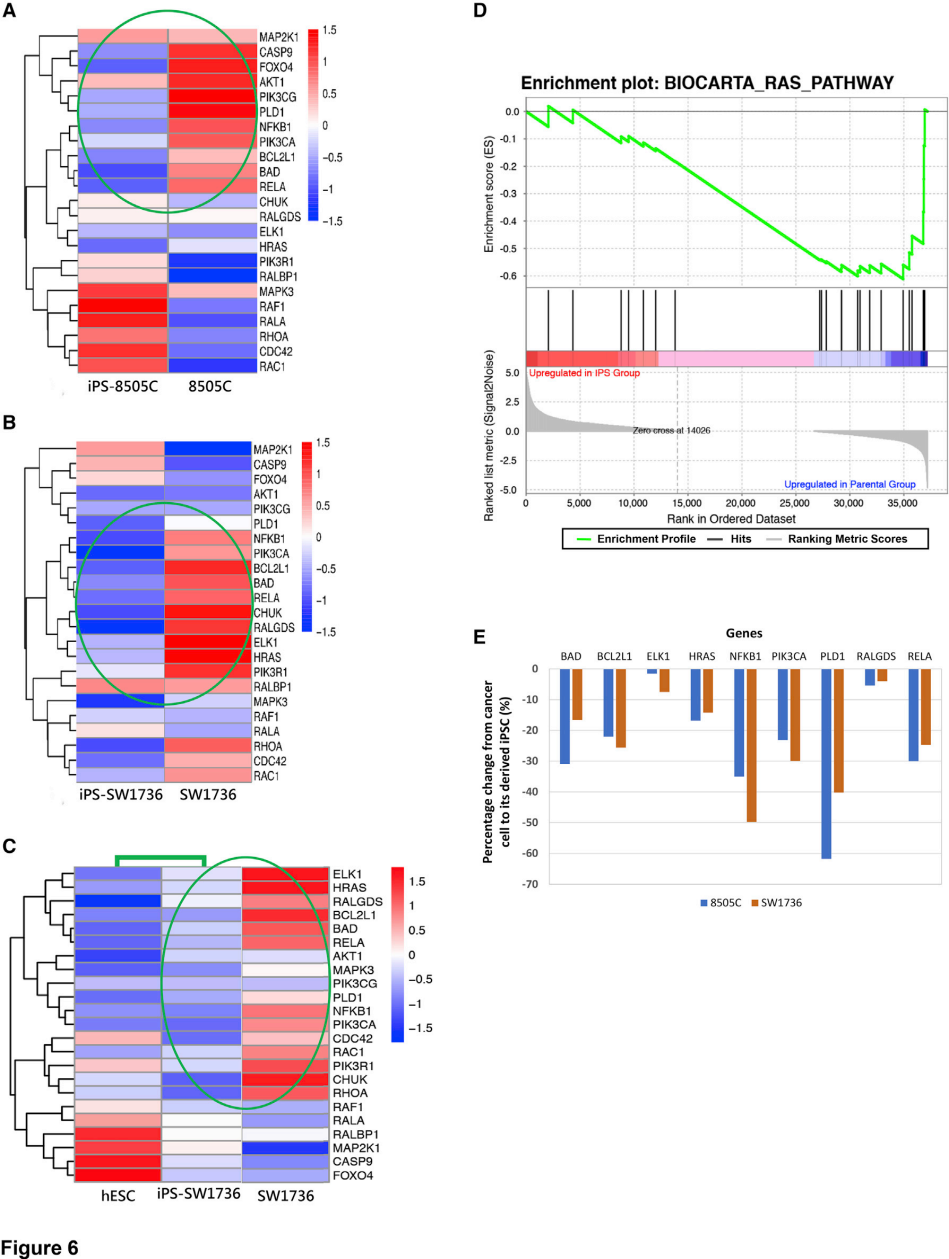
Key Oncogenic Components of the RAS Pathway Are Downregulated after Reprograming Cancer Cells to hiPSCs (A–C) Heatmaps of the cancer-derived hiPSCs (iPS-8505C) (A) and (iPS-SW1736) (B) compared with their corresponding parental cancer cells (8505C) (A) and (SW1736) (B) with respect to Biocarta RAS pathway genes. Most oncogenic RAS genes in the parental cancer line (seen as red) have been downregulated (seen as blue) in the hiPSCs to a level that now more closely emulates those of the hESCs (C). Most striking differences indicated by green ovals. Data that the cancer-derived hiPSCs cluster with hESCs on a global transcriptional level are provided in Figure 4. (D) Gene set enrichment analysis for the Ras signaling pathway dataset Biocarta Ras pathway showing that fewer genes in this pathway were expressed in the hiPSCs (grouping on the right side of the graph) compared with cancer cells (grouping on the left side). This analysis suggested that Ras signaling pathway-related genes were downregulated in hiPSCs. (E) Percentage decrease in the expression of representative onco-promoting genes in the RAS signaling pathway in the parental cancer cells (8505C and SW1736) when they were converted to hiPSCs. Here, neoplastic cells are very different from hESCs; the hiPSCs cluster closer to hESCs than to their neoplastic parent cells.

As noted above, of the four ATC lines tested, 8505C and SW1736 have a mutation in the Ras pathway—the V600E locus in the *BRAF* gene—whereas C643 and Hth74 do not (see Supplemental Information). Instructively, 8505C and SW1736 were the only ATCs that could be reprogrammed. And these ATC-derived hiPSCs could be maintained only in the presence of PD0325901, which specifically downregulates RAS signaling. Therefore, we next focused on the expression of genes involved in RAS signaling as likely being pivotal to the process of developmental regression, which, in essence, is what reprogramming to a normal epiblast-emulating hiPSC implies. Aided by the molecular signatures database with gene set enrichment analysis software (Figure 6), we generated heatmaps and Biocarta plots which showed that the ATC-derived hiPSC clones had gene expression patterns in the RAS pathway that were distinct from their parental cancer cells (Figures 6A and 6B).

Notably, key components of the RAS signaling cascade known to be most associated with oncogenesis and onco-propagation in the starting parental cancer line—seen as red in Figures 6A–6C—have strikingly been downregulated (green ovals) as these neoplastic cells were reprogrammed to “normality” (Figures 6D and 6E)—more closely emulating what their levels should be in early embryogenesis as modeled by hESCs (Figure 6C) (Altshuler et al., 2018; Ferreiros et al., 2019), including: *HRAS*; *ELK1* (a member of the Ets family of transcription factors and a nuclear target for the RAS-RAF-MAPK signaling cascade); *RELA* (“reticuloendotheliosis viral oncogene homolog A,” a proto-oncogene that belongs to the RHD/IPT transcription factor family and forms a heterodimer with nuclear factor κB [NFkB1]); *NFkB1* itself*; BAD* (BCL2-associated agonist of cell death)—to name a few. The Biocarta RAS pathway gene plot and its downstream gene enrichment plot further showed not only that expression of the genes related to the oncogenic components of the RAS pathway were lower in hiPSCs, but also that they clustered closer to hESCs than to their neoplastic parent cells (Figure 6D). Most of the RAS pathway oncogenes were downregulated in the hiPSCs (Figure 6E).

The use of an exquisitely specific pharmacological inhibitor of RAS signaling allowed us to examine conditional RAS expression within the same (i.e., isogenic) clone over time. The ability for relatively rapid initiation, then termination, then re-initiation of RAS signaling gave us an ideal experimental tool for fulfilling “Koch's postulates” without the potential for being confounded by the additional genetic manipulations required for genome editing (which is slow, protracted, polyclonal, and at risk for introducing off-target effects). We observed that any weakening of the RAS inhibition by withdrawal of PD0325901—allowing RAS pathway activity to rise again—not only blocked reprogramming but also allowed cancer to re-emerge from those same ATC-derived hiPSC clones; i.e., the hiPSCs converted back to neoplastic cells with the molecular patterns described above (Figures 3, 4, and 5). Interestingly, the cancer that re-emerged (in an unbiased, unprimed manner) from this presumably “clean slate” remained lineage specific. In other words, although RAS pathway aberrations have been associated with other cancer types—e.g., melanoma—even when starting from this “epiblast-like state,” in which all pluripotent ICM cells and then all primitive germ layer-resident cells bore the same genetic profile—only thyroid cancer reappeared; melanoma cells, for example, were not apparent in this isogenic preparation. This process of relieving RAS suppression in our hiPSCs and observing the emergence (or re-emergence) of neoplasia from a normal epiblast, provides a unique opportunity to scrutinize the process of lineage, tissue, and organ specification of a tumor from its pre-malignant state (although dissecting the many discrete elements of that process is beyond the scope of this present set of proof-of-concept experiments to proffer the reprogram enablement assay).

## Discussion

In this study, we report what we have learned about both the failure and the requirements for successful creation of normal epiblast-emulating cells (i.e., hiPSCs) from ATCs (the first from this virulently malignant cancer), suggesting that cancer-derived hiPSCs may, indeed, provide a tool for better understanding and targeting the earliest origins and progression of tissue-specific cancers (Camara et al., 2016; Pan et al., 2017)—an assay we have termed reprogram enablement. Furthermore, ICM-like cells derived from neoplastic cells, although ostensibly normal, do maintain—in a quiescent manner poised to be experimentally “unleashed”—the oncogenic potential of the starting cancer cells, enhancing this tool's utility. While it is true the final tumor seen in adults is an accumulation of multiple stepwise mutations, we have the opportunity here to “go back to square one” to ascertain the earliest pivotal molecular aberration—before the others “pile on” or perhaps to see the aberration from which the other abnormalities derive either directly or indirectly.

Virulently neoplastic cells from the thyroid could be reprogrammed to emulate cells of the normal human epiblast—based on a shift in the transcriptional, epigenetic, protein, differentiation, and functional profile of the ATC away from that of a neoplasm toward that of the ICM (as modeled by hESCs)—if and only if RAS signaling was tonically suppressed. While CD133^+^/CD44^+^ CSCs may have been present in the starting ATC population, they were undetectable in the cancer-derived hiPSCs, indicating that we were not simply witnessing a selection, expansion, and enrichment of CSCs present in the parental cancers. Furthermore, the gene expression profile, methylation landscapes, and principal component analyses of these ATC-derived hiPSCs clustered with hESCs, a pattern distinct from that of CSCs. Finally, in the presence of RAS suppression, the hiPSCs did not spawn ATCs (the functional definition of a CSC).

We hasten to add that these observations are not saying that perturbations in RAS are the only, or even the most lethal, genetic abnormalities in ATC. Indeed, by the time one excises and profiles an ATC from an adult patient—as was the case in the starting cancer cells used here—there are numerous aberrant genes and pathways noted. Which of these array of genetic abnormalities is most virulent is uncertain without extensive additional manipulations. However, the relatively rapid and simple assay we used here simply served to cast RAS signaling into relief as being the most prominent and pivotal at the earliest stages of oncogenesis, perhaps the genetic defect from which the others then cascade. It is likely that the genetic pathway(s) rendered salient will vary when this assay is applied to other cancer types from other organ systems.

The *BRAF* mutation present in the two successfully reprogramed ATCs can be found in ∼55% of advanced thyroid cancers, most commonly a valine-to-glutamic acid substitution at residue 600 (p.V600E) (Santarpia et al., 2008). Mutated BRAF can influence kinase activity by constitutively activating MEK and ERK (Li et al., 2017; Marusiak et al., 2014). That our successful complete reprogramming of ATC back into normal human epiblast-emulating cells is likely dependent on suppressing RAS signaling is complementary to and builds upon older findings on the critical role of properly reduced RAS activity for normal early embryonic development (Altshuler et al., 2018; Ferreiros et al., 2019). Furthermore, the neoplasia of the 8505C and SW1736 ATC cells are dependent on RAS activation; we were clearly repressing a pathway that blocks reprogramming—i.e., that inhibits developmental regression to a normal pre-malignant embryonic epiblast-like state. (The ATCs also have a genetic variation in the *HRAS* gene, specifically in codon 27 of exon 1 of *HRAS* [His27His] [see Supplemental Information], which is known epidemiologically to correlate with cancer risk [Wang et al., 2017a]; however, this is a silent polymorphism and does not alter the HRAS protein sequence nor the RAS activity, pointing to the *BRAF* mutation as the cause of the abnormally increased RAS pathway activity.)

Critically, key components of the RAS signaling cascade known to be the most oncogenic in the starting parental cancer line (seen as red in Figures 6A–6C) have strikingly been downregulated as the neoplastic cells are reprogrammed to normality, more closely emulating what their levels should be in early embryogenesis (as modeled by hESCs). These genes include (detailed under Results): *HRAS*, *ELK1*, *RELA*, and *NFkB1*—to name just a few.

The use of a rapidly acting and reversible, exquisitely specific pharmacological inhibitor of RAS signaling allowed us to examine conditional RAS expression within the same (i.e., isogenic) clone over time—lack of expression versus re-expression (with few, if any, confounders). Any weakening of RAS suppression by withdrawal of the inhibitor—permitting RAS pathway activity to rise again—not only blocked reprogramming but also enabled cancer to re-emerge from those same ATC-derived hiPSC clones, i.e., for the hiPSCs to convert back to neoplastic cells, with the genetic patterns described in Figures 3, 4, and 5. This process in itself—experimentally relieving RAS suppression in our ATC-derived hiPSCs, allowing one to watch the emergence (or re-emergence) of neoplasia from a normal epiblast—provides a unique opportunity for scrutiny and manipulation of the earliest stages of oncogenesis. Our success in converting the same cancer cell to a normal epiblast-like cell and then back to a cancer cell again allowed us to recognize the pivotal role of excessive RAS signaling—perhaps more so than any other aberration—in the earliest emergence of thyroid cancer; “RAS suppression” is ATC's reprogram enablement factor. Presumably, different pathways will become salient when this approach is applied to other cancer types.

Interestingly, although RAS aberrations have certainly been associated with neoplasms other than thyroid cancer (e.g., melanoma), the cancer that re-emerged—in an unbiased non-primed fashion from the presumably clean slate we created—remained lineage specific. In other words, even when starting from the epiblast-like state in which all pluripotent ICM-like cells and then all primitive germ layer-resident cells bore the same genetic profile, only thyroid cancer reappeared; no melanoma cells, for example, were apparent in this isogenic preparation. This system should now allow the process of such lineage, tissue, and organ specification of a tumor in its pre-malignant state to be studied.

With regard to the thyroid cancer cells that carried a wild-type *BRAF* gene yet still could not be reprogrammed (C643 and Hth74), an entirely different blocking pathway is clearly operative—suggesting, importantly, that the old categorization scheme for designating ATCs based on histopathological characteristics may actually need to be updated, refined, and rendered more discrete based, at least in part, on the functional and molecular attributes unveiled by the reprogram enablement assay.

This kind of approach can also provide NGCMs for drug development. While more cancer samples need to be studied to further understand the function of major pathways in determining the fate of cells from different genetic backgrounds and tissues-of-origin, we believe the strategy we used for ATCs can be applied to other types of neoplasia.

This broadly applicable strategy of pushing a single clone back to its normal epiblast-emulating state—learning empirically the earliest pivotal “master regulatory” pathways that abet or obstruct that reprogramming process—and watching cancer re-emerge in that starting clone versus going down multiple clonally related isogenic lineages—can be used in conjunction with other NGCMs. For example, this approach represents the “flip-side” of the type of study recently published in which normal hiPSCs were transformed into cancer by inserting candidate genes culled from The Cancer Genome Atlas of known oncogenes (Koga et al., 2020). Coupling such studies with ours—wherein we do not start with normal hiPSCs but rather determine what it takes to transit cancers back to being normal hiPSCs (i.e., to their normal embryonic origins)—may help more efficiently suggest or rank order the genes that do (or should) populate such databases going forward. Such studies, when paired with ours, may confirm key oncogenic targets for early or even pre-emptive therapy for a given tissue-specific neoplasm.

## Experimental Procedures

Details of reprogramming, iPSC characterization, gene expression, and epigenetic analysis, including vendors and catalog numbers, are provided in the Supplemental Information.

## Author Contributions

Y.K. designed and performed the experiments, interpreted the data, and wrote the manuscript. R.C.G. and R.N.McV. assisted with obtaining and interpreting data. Y.L. assisted with designing, performing, and interpreting the experiments, writing the manuscript, and providing supervision. A.P.H. and J.Y. performed bioinformatics analysis. W.Z. assisted with experimental design and supervision. E.Y.S. provided overall supervision of experimental design, data acquisition and interpretation, manuscript writing, and provided funding.

## Data and Code Availability

The Gene Expression Omnibus accession number for the RNA-seq and MethylSeq data reported in this paper is GEO: GSE154280.

## Supplemental Information

Document S1. Supplemental Experimental Procedures, Figures S1–S4, and Table S1

Document S2. Article plus Supplemental Information

## Footnotes

Supplemental Information can be found online at https://doi.org/10.1016/j.stemcr.2020.07.016.
