# Supplementary material for: “Reprogram Enablement” as an Assay for Identifying Early Oncogenic Pathways by Their Ability to Allow Neoplastic Cells to Reacquire an Epiblast State
Source: Stem Cell Reports. 2020 Aug 13;15(3):761–75. doi: 10.1016/j.stemcr.2020.07.016 (PMC7486218; doi:10.1016/j.stemcr.2020.07.016)
Supplement: Document S1. Supplemental Experimental Procedures, Figures S1–S4, and Table S1 [file mmc1.pdf]

Stem Cell Reports, Volume *15*

Supplemental Information

"Reprogram Enablement" as an Assay for Identifying Early Oncogenic Pathways by Their Ability to Allow Neoplastic Cells to Reacquire an Epiblast State

Yanjun Kong, Ryan C. Gimple, Rachael N. McVicar, Andrew P. Hodges, Jun Yin, Yang Liu, Weiwei Zhan, and Evan Y. Snyder

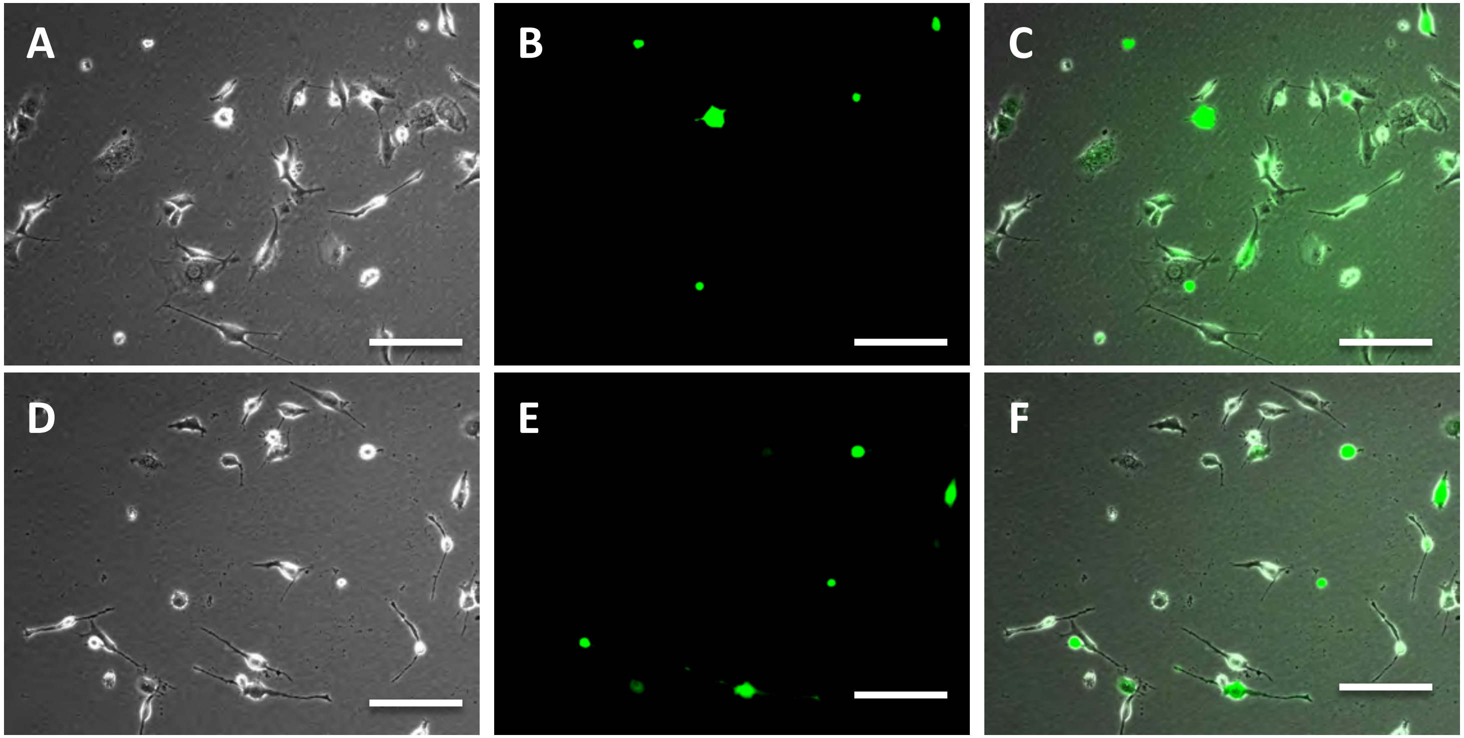

Figure S1

FIGURE S1: Electroporation efficiency of episomal transfection. (Related to Figs. 1A-E and 2A-D)

[A] A photomicrograph under bright field of ATC line 8505C. **[B]** GFP **(green)** expression in 8505C cells after co-electroporation with episomal vectors carrying the OSKM reprogramming factors. **[C]** Overlay of **[A]** & **[B]**. Scale bars: 100 µm.

[D] A photomicrograph under bright field of ATC line SW1736. **[E]** GFP expression in SW1736 cells after co-electroporation with the episomal vectors carrying the OSKM reprogramming factors. **[F]** Overlay of **[D]** & **[E]**. Scale bars: 100 µm.

In both cases, the percentage of green cells indicates the efficiency of likely transfection.

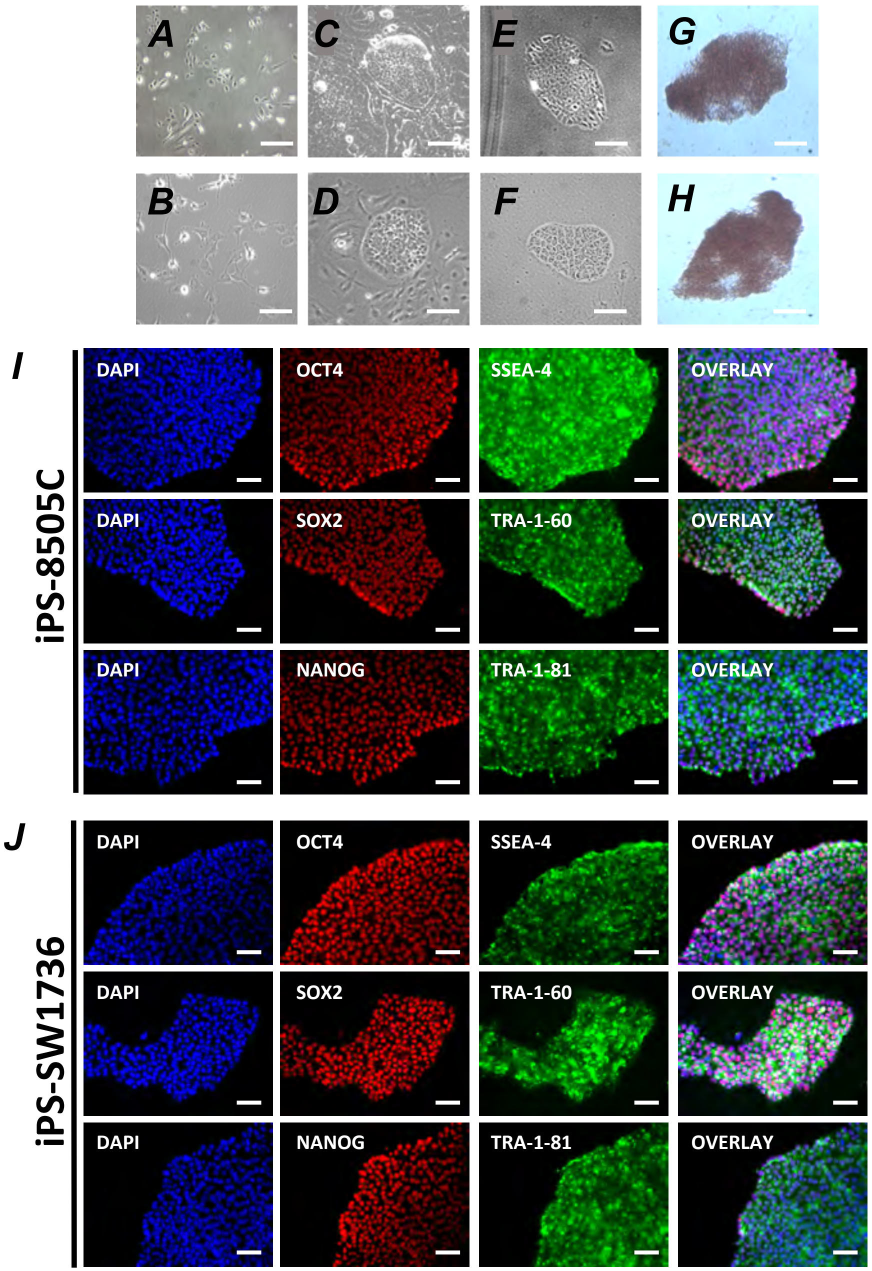
A
B
C
D
E
F
G
H
I
iPS-8505C
DAPI
OCT4
SSEA-4
OVERLAY
DAPI
SOX2
TRA-1-60
OVERLAY
DAPI
NANOG
TRA-1-81
OVERLAY
J
iPS-SW1736
DAPI
OCT4
SSEA-4
OVERLAY
DAPI
SOX2
TRA-1-60
OVERLAY
DAPI
NANOG
TRA-1-81
OVERLAY

Figure S2

FIGURE S2: Confirmation of the pluripotency of the cancer-derived hiPSC clones based on morphology and marker expression. (Related to Fig. 1A)

Morphology of parental cancer (ATC) cell lines **[A]** 8505C and **[B]** SW1736. Morphology of the hiPSC lines **[C]** iPS-8505C and **[D]** iPS-SW1736 before isolation. Morphology of a colony of **[E]** iPS-8505C and **[F]** iPS-SW1736 after 10 passages, resembling normal hESC colonies. Live staining of an **[G]** iPS-8505C colony and **[H]** iPS-SW1736 colony expressing alkaline phosphatase **(reddish-brown)**. *Pluripotency marker immunochemical staining* of **[I]** iPS-8505C and **[J]** iPS-SW1736. SSEA-4, Tra-1-60, and Tra-1-81 are surface markers; OCT-4, SOX2, and NANOG are nuclear markers. Scale bars: 100 μm.

See **Fig. 1A** for pluripotency gene expression.

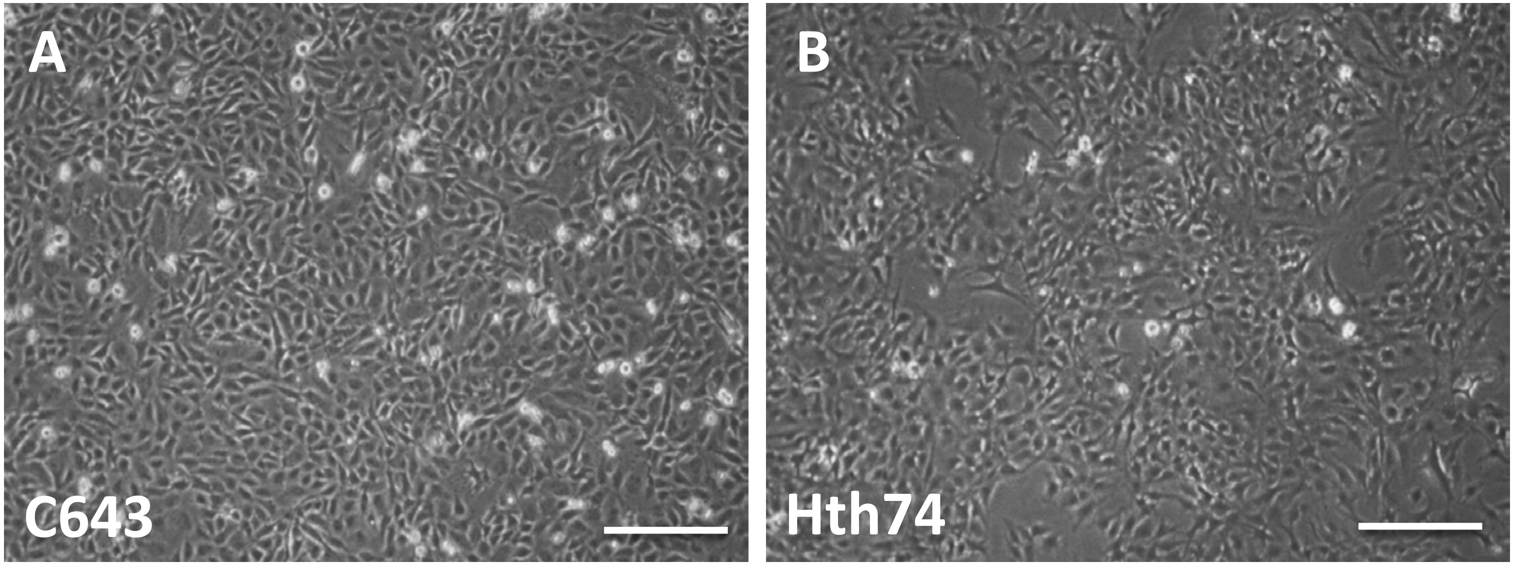

Figure S3

FIGURE S3: No hiPSC colonies were observed in the ATC lines C643 and Hth74 (i.e., those without a BRAF mutation), regardless of the reprogramming methods or small molecules employed. (Related to Figs. 1C, D)

[A] No colonies emerged after reprogramming with different methods from ATC line C643. **[B]** Similarly, no colonies emerged after reprogramming with different methods from ATC line Hth74. Only ATC cancer cells persisted, as shown. Scale bars: 100 µm.

See **Figs. 1C, D** and **Fig. S2** for images of successful hiPSC colony formation.

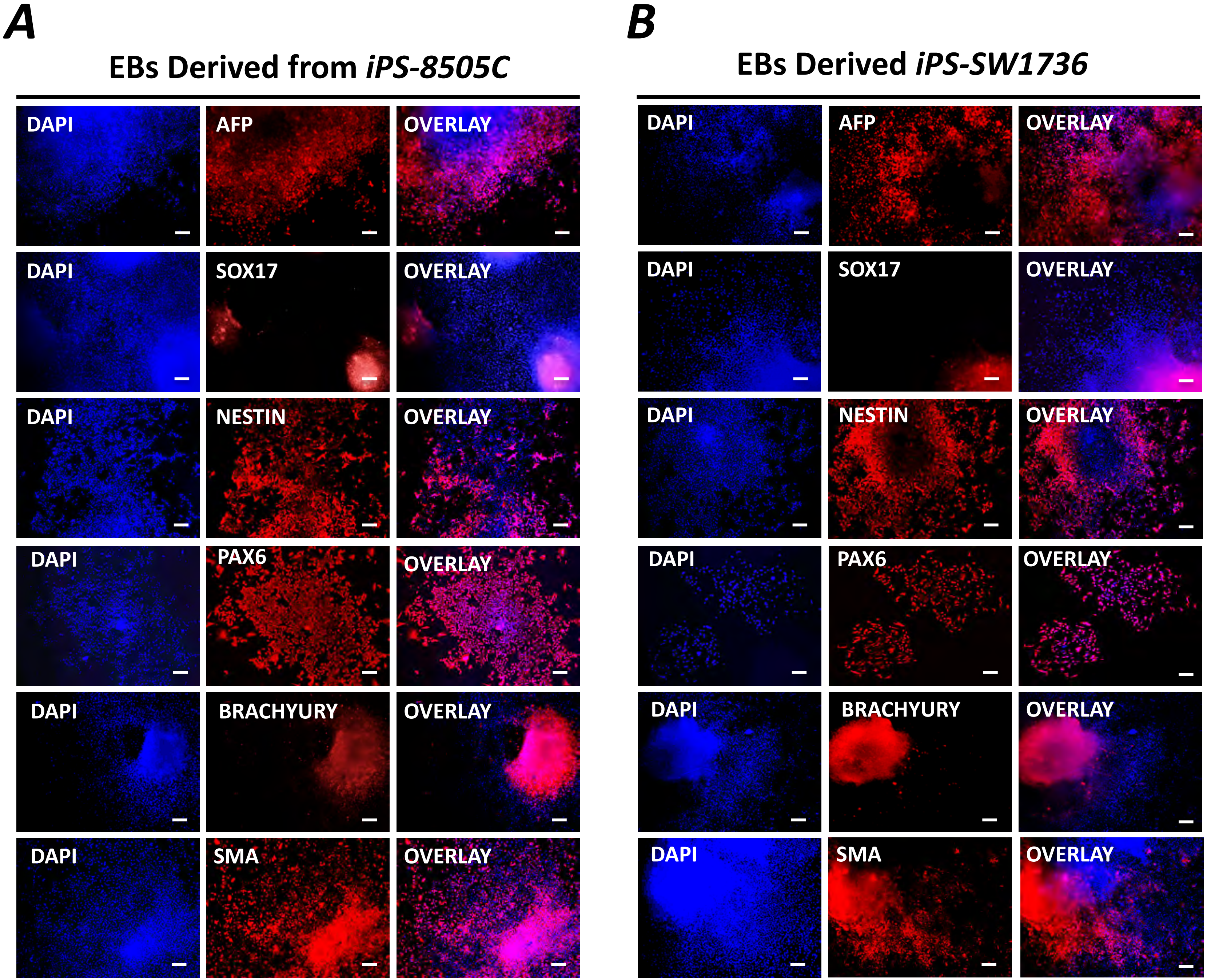
A
EBs Derived from iPS-8505C
DAPI
AFP
OVERLAY
DAPI
SOX17
OVERLAY
DAPI
NESTIN
OVERLAY
DAPI
PAX6
OVERLAY
DAPI
BRACHYURY
OVERLAY
DAPI
SMA
OVERLAY
B
EBs Derived iPS-SW1736
DAPI
AFP
OVERLAY
DAPI
SOX17
OVERLAY
DAPI
NESTIN
OVERLAY
DAPI
PAX6
OVERLAY
DAPI
BRACHYURY
OVERLAY
DAPI
SMA
OVERLAY

Figure S4

FIGURE S4: Differentiation of hiPSCs into lineages representative of each of the 3 fundamental germ layers which emerged spontaneously when they were permitted to form epiblast "organoids", also termed "embryoid bodies" (EBs). (Related to Figs. 2A, B)

Sub-populations of iPS-8505C **[A]** and iPS-SW1736 **[B]** cells within their respective EBs stopped expression pluripotency markers and started immunostaining for alpha fetal protein (AFP) and SOX17 (endoderm markers); PAX6 and, later, NESTIN (ectoderm, specifically neurectoderm, markers); Brachyury and smooth muscle actin (SMA) (mesoderm markers). Scale bars: 100 μm.

See **Fig. 2A, B** for measures of gene expression.

SUPPLEMENTAL TABLES

TABLE S1: Methylation analysis at the SOX2 and SALL4 loci. (Related to Fig. 5E)

TABLE S1: *Methylation analysis at the SOX2 and SALL4 loci**

Chromosome	Start	End	p-Value	q-Value	Meth Diff	Gene Name
chr3	181431315	181431315	1.07E-34	2.10E-31	-94.444444	SOX2
chr3	181431298	181431298	9.86E-30	9.54E-27	-88.333333	SOX2
chr3	181430369	181430369	1.07E-23	4.14E-21	-96	SOX2
chr3	181431309	181431309	1.57E-23	5.91E-21	-75.471698	SOX2
chr3	181430435	181430435	6.13E-23	2.12E-20	-75.675676	SOX2
chr3	181431307	181431307	7.33E-23	2.49E-20	-75.471698	SOX2
chr3	181428236	181428236	1.51E-20	3.67E-18	-65.789474	SOX2
chr3	181430403	181430403	1.90E-18	3.39E-16	-69.69697	SOX2
chr3	181430422	181430422	5.34E-18	8.97E-16	-65.714286	SOX2
chr3	181430397	181430397	7.36E-17	1.05E-14	-60.526316	SOX2
chr3	181430466	181430466	6.03E-16	7.55E-14	-62.5	SOX2
chr3	181433606	181433606	5.62E-14	5.33E-12	-65.384615	SOX2
chr20	50419083	50419083	1.30E-70	1.32E-65	-89.188596	SALL4
chr20	50419043	50419043	1.86E-69	1.54E-64	-95.652174	SALL4
chr20	50419045	50419045	4.27E-66	1.95E-61	-91.752577	SALL4
chr20	50419034	50419034	1.23E-65	5.34E-61	-96.470588	SALL4
chr20	50419038	50419038	1.65E-64	6.30E-60	-92.391304	SALL4
chr20	50419232	50419232	8.38E-45	5.63E-41	-94.827586	SALL4
chr20	50419194	50419194	1.06E-41	4.84E-38	-90.322581	SALL4
chr20	50418996	50418996	1.76E-41	7.71E-38	-91.37931	SALL4
chr20	50418977	50418977	6.52E-39	2.16E-35	-97.959184	SALL4
chr20	50419215	50419215	8.44E-36	1.99E-32	-91.308382	SALL4
chr20	50418960	50418960	1.57E-35	3.63E-32	-95.652174	SALL4
chr20	50419249	50419249	3.30E-35	7.26E-32	-84.210526	SALL4
chr20	50418968	50418968	1.93E-34	3.96E-31	-93.478261	SALL4
chr20	50418938	50418938	1.15E-33	2.14E-30	-90.148148	SALL4
chr20	50418943	50418943	2.14E-32	3.45E-29	-88.815526	SALL4
chr20	50418891	50418891	1.20E-31	1.79E-28	-89.37037	SALL4
chr20	50419269	50419269	1.20E-31	1.79E-28	-87.912088	SALL4
chr20	50418907	50418907	1.94E-29	2.14E-26	-83.514386	SALL4
chr20	50418965	50418965	1.98E-29	2.19E-26	-88.372093	SALL4
chr20	50419349	50419349	4.06E-29	4.35E-26	-76.983081	SALL4
chr20	50418880	50418880	4.36E-29	4.67E-26	-89.450355	SALL4
chr20	50418928	50418928	7.92E-28	7.22E-25	-83.071769	SALL4
chr20	50418953	50418953	1.35E-23	6.86E-21	-71.73913	SALL4
chr20	50419275	50419275	4.90E-21	1.76E-18	-68.367347	SALL4
chr20	50418865	50418865	1.98E-19	5.66E-17	-74.358974	SALL4
chr20	50418850	50418850	8.56E-19	2.22E-16	-91.666667	SALL4
chr20	50414840	50414840	4.81E-18	1.12E-15	-62.397554	SALL4
chr20	50418120	50418120	8.14E-09	4.73E-07	-64	SALL4

*****See Figure 5E

SUPPLEMENTAL EXPERIMENAL PROCEDURES

1. Cell culture

ATC lines C643, Hth74, 8505C and SW1736 were purchased from University of Colorado Cancer Center (University of Colorado Anschutz Medical Campus, USA). Their *genetic background* is as listed here.

**Cell Line**	**Mutation Type**					
	**BRAF**	**TP53**	**HRAS**	**RET**	**CTNB B**	**TERT**
8505C	V600E	R248	H27H	WT	WT	Heterozygous at c.250 C>T (-146 C>T); in promoter
SW1736	V600E	NA*	NA*	WT	NA*	Heterozygous at c.228 C>T (-124 C>T); in promoter
C643	WT	R248	G13R	WT	WT	Heterozygous at c.228 C>T (-124 C>T); in promoter
HTh74	WT	NA*	H27H	WT	WT	Heterozygous at c.228 C>T (-124 C>T); in promoter

* not reported; WT = wild type (normal)

The cells were cultured in RPMI 1640 medium (Invitrogen) supplemented with 10 % FBS (Hyclone, Cat# SV30014.03), 1% Anti-Anti ((Life Technologies, Cat# 15240-062), 1% GlutaMAX (Gibco, Ref: 35050-061) at 37ºC in a 5% CO_2-air incubator. hESC line H1 (NIH catalogue # WA01) was cultured (as previously described in Lee et al, 2007) in mTeSR^{TM} media (Stem Cell technologies, Cat# 85870) with 0.1mg/ml Matrigel (Corning, Cat# 356231) coated plates at 37ºC in a 5% CO_2-air incubator. Cells were passaged with Accutase solution (Millipore sigma, Cat# SCR005) and media was changed on a daily basis.

2. Reprogramming

2.1 SeV-mediated reprogramming

Sendai virus (SeV) vectors encoding reprogramming factors OCT4, SOX2, c-MYC and KLF4 were purchased from Invitrogen (Cat# A16518). The viral transduction was performed based on manufacturer's instructions. The expression level of the stemness genes in cancer cell lines was determined by quantitative polymerase chain reaction (qPCR) [. Thyroid tumor cells were plated at a density of $2x10^5$ cells/well of a six-well plate in cancer cell culture medium and kept overnight before the transduction. The conventional hiPSC generation SeV vector cocktail (containing polycistronic-KLF4, OCT4, and SOX2; monocistronic-c-MYC, and monocistronic-KLF4 was transduced to the cells at multiplicity of infection (MOI) of 5:5:3, respectively. After transduction, the cells were maintained in the same culture condition and morphology change was observed every day. 5 days after transduction, the cells were transferred to Matrigel-coated plates with half RPMI medium and half mTeSR medium. The next day the medium was changed to mTeSR. The cells were grown in mTeSR for 30-45 days.

hiPSCs were maintained as previously described by us in Tobe et al, 2017.

After 4-6 weeks post-induction, clones with hESC/hiPSC morphology were visualized microscopically. Live staining was performed with TRA-1-60 mouse anti-human mAb, AlexaFluor 488 Conjugate Kit for live Cell Imaging (Life technology, Cat. no. A25618) according to the manufacture instruction. Then hiPSC-like colonies were manually transferred into 24 well plates. Reprogramming efficiency was determined by the percentage of the cells that were triple positive for stem cell markers Tri1-

81, SSEA-4 and Tri-1-60 using the flow cytometry analysis.

2.2 Episomal vector transfection

The cancer cells were transfected with the Episomal DNA cocktail at 1ug/uL: pCXLE-Oct4/shP53 (Addgene, Cat# 27077); pCXLE-Sox2/Klf4 (Addgene, Cat# 27078); pCXLE-LMyc/Lin28 (Addgene, Cat# 27080); pCXLE-EGFP (Addgene, Cat# 27082) using the Neon Electroporation System (Life Technologies, Cat # MPK5000). The transfected cells were cultured in the same way as Sendai virus transduction.

Integration of reprogramming genes – whether transfected by SeV or episome (which are regarded as non-integrating vectors) -- was routinely ruled out by PCR.

2.3 Small molecule treatment

The following small molecules were purchased from LabEaze and added after reprogramming to all the four cell lines SW1736, 8505C, Hth74 and C643:

Small Molecule	Function	Targeted Signaling Pathway
Y-27632	Rho-associated kinase (ROCK) family inhibitor	ROCK/Rho signaling
SB431542	Potent and selective inhibitor of TGF-β R1 (ALK5)	Transforming Growth Factor-β (TGF-β) Signaling
CHIR99021	GSK-3α and GSK-3β inhibitor	PI3K/Akt/mTOR Signaling
PD0325901	Selective and non ATP-competitive MEK inhibitor	RAS/RAF/MAPK Signaling

The respective concentrations and combinations with which they were tested as post-reprogramming culture supplements is shown below:

Small Molecule	Code	Concentration (μM)	Combinations Tested
Y-27632	A	1, 5*, 10*, 15, 20	AB, AC, AD, BC, BD, CD, ABC, ABD, ACD, BDC, ABCD
SB431542	B	1, 2*, 4*, 8, 16	
CHIR99021	C	1, 2*, 4*, 8, 16	
PD0325901	D	0.25*, 0.5*, 1, 2, 4	

*doses used for different drug combinations

Note that, once RAS pathway suppression proved to be an important variable (indeed, an existential factor), we used such pharmacologic RAS pathway inhibitors during hiPSC generation, maintenance, and, for consistency, and as a control in all samples for all assessments described below and throughout the study (including in comparisons with parental cancer lines).

3. **Characterization of hiPSCs**

3.1 Sendai virus antibody test

Immunocytochemistry (ICC) was performed using routine procedures with polyclonal anti-Sendai virus antibody (MBL, Cat# D029)

3.2 DNA fingerprinting/STR

Total DNA was collected from cells using DNeasy Blood & Tissue Kit (Qiagen, cat. no. 69504). DNA concentration was tested using Nanodrop2000 (Thermo scientific). Short tandem repeat (STR) DNA profiling analysis was done by PowerPlex® 16 HS (Promega Corporation) with highly informative and unambiguous Multiplex STR technology. Fifteen autosomal loci and 1 gender determination marker were tested. Then the DNA Profile of the 16 Genetic Sites (D8S1179, D21S11, D7S820, CSF1PO, D3S1358, TH01, D13S317, D16S539, vWA, TPOX, D18S51, D5S818, FGA, Amelogenin, Penta D, Penta E) were compared with the reference ECACC cell line online database.

3.3 Alkaline Phosphatase (AP) Staining Assay

The activity of AP, a marker of undifferentiated pluripotent hESCs and hiPSCs, was used to confirm the pluripotency of the ATC-derived hiPSCs. Staining was done with the BPStem™ Alkaline Phosphatase Staining Assay Kit (Catalog Number SC-003) according to the manufacturer's instructions. Cells were cultured in 24 well plates for 4 days. The medium was aspirated and the cells washed with 1xPBST containing 0.05% Tween-20. The cells were fixed with 0.4ml fixing solution at room temperature for 2 minutes. The fixative was aspirated and the fixed cells washed with 1x PBST. The washing buffer was removed and 0.4ml Staining Solution added. The cells were incubated at room temperature for 20 minutes and then observed under an inverted microscope.

3.4 Immunocytochemistry (ICC) Staining

Cells were fixed with 0.5 ml 4% paraformaldehyde in PBS for 20 min and blocked with 5%BSA+0.1% Triton-100 for 1h at room temperature. Primary antibodies are purchased from cell signaling, Nanog (D73G4) XP® Rabbit mAb #4903, TRA-1-81 (TRA-1-81) Mouse mAb #4745,TRA-1-60(S) (TRA-1-60(S)) Mouse mAb #4746,SSEA4 (MC813) Mouse mAb #4755,Oct-4A (C52G3) Rabbit mAb #2890. Primary antibodies were diluted in 5%BSA and primary antibodies' incubation were done at 4ºC overnight. The Primary antibodies for EB characterize were PAX6, Abcam Donkey Anti-Mouse IgG H&L (Alexa Fluor® 488) (ab150105) and Donkey Anti-Rabbit IgG H&L (Alexa Fluor® 555) (ab150074) were used as secondary antibodies. Cells were stained with the nuclear marker DAPI (Vector) for 10 mins to visualize all cells in the field and then examined using a fluorescence microscope (Nikon).

3.5 Embryoid body (EB) ("epiblast organoid") formation and spontaneous differentiation

hiPSCs were dissociated manually when at 90% confluence and plated on ultra-low attachment plates (Corning, Cat# 3471). The cells were cultured in DMEM/F12 (Life Technologies, Cat# 11330-057) supplemented with 20% Knockout™SR (Gibco, Cat# 10828-028), 1% GlutaMAXTM-1 (Gibco, Cat# 35050-061), 1% MEM Non-Essential Amino Acid (NEAA) (Life Technologies, Cat# 11140-050) and 0.2% 2-Mercaptoethanol (Gibco, Cat# 21985-023). Various sized spheroids could be observed on the second day. Medium was changed every other day. EBs were collected on day 10 and transferred to 0.1% gelatin coated plate culturing for another 10 days. Then the cells were detached with Accutase for the qPCR analysis.

3.6 RNA Preparation and Quantitative real-time PCR

Total RNA was extracted from cells and EBs using RNeasy Mini Kit (Qiagen, Cat# 74104). Reverse transcription was performed by SuperScript III First-Strand Synthesis SuperMix for qRT-PCR (Invitrogen, Cat# 11752). Quantitative real time RT-PCR analysis was performed with SensiFAST SYBR ® No-ROX Kit (Bioline, Cat# BIO-98005). All gene expression levels were normalized against glyceraldehyde-3-phosphate dehydrogenase (GAPDH) mRNA expression. The primers for pluripotency genes and early differentiation were designed based on sequences on GenBank (PubMed).

The primers used are shown in the accompanying Table below. Each gene expression experiment contained 3 repeats.

**Primer Name**	**Sequence**
hOCT3/4-S	AGC AAA ACC CGG AGG AGT
hOCT3/4-AS	CCA ACT CGG CCT GTG TAT ATC
hSOX2-S	CTC CGG GAC ATG ATC AGC
hSOX2-AS	CTG GGA CAT GTG AAG TCT GC
hNANOG-S	CTC CAT GAA CAT GCA ACC TG
hNANOG-AS	CTC GCT GAT TAG GCT CCA AC
hLIN28-S	CTG TCC AAA TGC AAG TGA GG
hLIN28-AS	GCA GGT TGT AGG GTG ATT CC
hKLF4-S	ATT GGA CCC GGT GTA ACT TC
hKLF4-AS	AGC ACG AAC TTG CCC ATC
hc-MYC-S	CAC CAG CAG CGA CTC TGA
hc-MYC-AS	GAT CCA GAC TCT GAC CTT TTG C
hGAPDH-S	AGC CAC ATC GCT CAG ACA C
hGAPDH-AS	GCC CAA TAC GAC CAA ATC C
hSOX17-S	CGC TTT CAT GGT GTG GGC TAA GGA CG
hSOX17-AS	TAG TTG GGG TGG TCC TGC ATG TGC TG
hAFP-S	GAA TGC TGC AAA CTG ACC ACG CTG GAA C
hAFP-AS	TGG CAT TCA AGA GGG TTT TCA GTC TGG A
hBRACHYURY-S	CTC TCC CTC CCC TCC ACG CAC AG
hBRACHYURY-AS	GCG CCG TTG CTC ACA GAC CAC AGG
hMSX1-S	CGA GAG GAC CCC GTG GAT GCA GAG
hMSX1-AS	GGC GGC CAT CTT CAG CTT CTC CAG
haSMA-S	CTG TTC CAG CCA TCC TTC AT
haSMA-AS	CGG CTT CAT CGT ATT CCT GT
hPAX6-S	ACC CAT TAT CCA GAT GTG TTT GCC CGA G
hPAX6-AS	ATG GTA AAG CTG GGC ATA GGC GGC AG
hNESTIN-S	GGC GCA CCT CAA GAT GTC C
hNESTIN-AS	CTT GGG GTC CTG AAA GCT G

4. Cancer phenotype characterization

4.1 Proliferation Assay

To measure *cell proliferation* rate, we performed direct cell counting because we found it more sensitive than surrogate assays (e.g., resazurin, CellTiter-Glo [crystal violet], PicoGreen, Hoechst, or CyQuant,) for determining the number of viable cells.

Cells were cultured in 12 well plates and were collected and calculated with BIO-RAD Automatic cell counter and its slides (BIO-RAD, Cat# 145-0011) at four time points at 24, 48, 72, and 96 hours. hiPSCs were cultured with PD0325901 (LabEaze, Cat# SM001) throughout the test. Three repeats were done for each group.

4.2 Invasion and Migration Assay

Assay was performed according to corning cell migration, chemotaxis and invasion assay using staining protocol with 6.5mm Transwell with 8.0µm Pore Polycarbonate Membrane Insert plate (Corning, Cat# 3422). hiPSCs were cultured with PD0325901 (LabEaze, Cat# SM001) throughout the test. Before the test, cells were dissociated with Accutase and resuspended in phenol red free IMDM medium (Gibco, Ref: 21056-023). And 100 µl cell suspension were seeded in each insert. 650 µl IMDM with or without 10% FBS were used as control or the experiment group. An insert coated with Matrigel was used for the invasion assay and the non-coated ones were used for the migration analysis. Cells were incubated for 12 hours in 37ºC. They were then stained with 0.23% crystal violet dye and observed under an inverted microscope. Pictures of 4 different areas of the membrane on each insert membrane were taken by software QCapture 2.9.12 (Silicon Graphics, Inc) and cell number was counted with Image J (ImageJ 1.5 K, Wagne Rasband National Institutes of Health, USA). Studies for each group were performed in triplicate.

4.3 Cell viability

Plating density ($1x10^4$) and assay timing were optimized. Cells were seeded in a 96 well plate at a density of $1x10^4$ per well. The next day, the chemotherapeutic agent, doxorubicin (Selleckchem, Cat# S1208) or dimethylsulfoxide DMSO (DMSO) (ATCC, Part No: 4-X-5), as a negative control, was added to the medium, and culture for 48 hours. One group received just regular medium as a second negative control. hiPSCs were cultured with PD0325901 (LabEaze, Cat# SM001) throughout the test. Each group was tested in triplicates. Cell growth was determined using an MTT kit (Sigma, Stock No. CGD-1) to calculate the viability of the cells. MTT SOLUTION was added aseptically in an amount equal to 10% of the culture volume. A Perkin-Elmer Wallac EnVision 2103 Multilable Reader was used to measure absorbance at a wavelength of 570 nm.

5. Gene expression analysis

5.1 RNA-Seq and Methyl-Seq

Total RNA was extracted from cells with RNeasy Mini Kit (Qiagen, Cat# 74104). RNA-free DNA was isolated from cells with DNeasy Blood & Tissue Kit (Qiagen, Cat# 69504). The quality of DNA was tested by Qubit Fluorometer. Next Generation Sequencing was performed using an Illumina NextSeq 500 with TruSeq Methyl Capture EPIC Library Prep Kit and KAPA HiFi HotStart Uracil ReadyMix PCR Kit. Then the sequencing data was analyzed via Integrative Genomics Viewer (http://software.broadinstitute.org/software/igv/).

6. Statistical analysis

All data were shown as a mean with error bars representing SEM. Statistical analysis was performed according to the data type and test purpose: differences between two groups were analyzed using unpaired Student's t-test; differences and one-way ANOVA tests were used when three or four groups were compared. All p-values were based on two-tailed statistical analyses, and a p-value of <0.05 was considered the minimum for statistical significance; the following convention was used: * $p<0.05$; ** $p<0.01$; *** $p<0.001$). All tests were analyzed with GraphPad Prism 7.04 (GraphPad Software, La Jolla, CA, USA).
